# Acute coronary artery spasm and myocardial infarction induced by inhalational methanol poisoning: A case report

**DOI:** 10.1097/MD.0000000000044752

**Published:** 2025-09-19

**Authors:** Jinjun Li, Shu Zhou, Dun Ao

**Affiliations:** a Department of Emergency Medicine, Liuyang People’s Hospital, Liuyang, Hunan Province, China.

**Keywords:** acidosis, coronary artery spasm, methanol, myocardial infarction, vision disorders

## Abstract

**Rationale:**

Methanol poisoning is a rare but potentially fatal condition characterized by metabolic acidosis, neurological dysfunction, and visual impairment. Acute myocardial infarction caused by coronary artery spasm as a complication of methanol poisoning is extremely rare and has seldom been reported in the literature.

**Patient concerns:**

A 46-year-old male presented to the emergency department with acute chest pain, dyspnea, dizziness, and blurred vision lasting for 4 hours. He had no prior history of cardiovascular diseases but reported potential occupational exposure to methanol vapors during pyrotechnic bright bead production 2 days prior to symptom onset.

**Diagnoses:**

The patient demonstrated ST-segment elevation in the inferior leads on the electrocardiogram, accompanied by elevated cardiac biomarkers, indicative of an acute myocardial infarction. Furthermore, the patient presented with severe metabolic acidosis and visual disturbances, with a blood methanol concentration of 823 μg/mL, confirming a diagnosis of methanol poisoning.

**Interventions:**

Coronary angiography identified a total occlusion in the mid-segment of the right coronary artery, which was effectively managed through balloon angioplasty and the administration of intracoronary nitroglycerin. Continuous renal replacement therapy was commenced to rectify metabolic acidosis and facilitate the removal of methanol and its toxic metabolites. Fomepizole was not used due to regional unavailability and ethanol was not administrated due to its cardiovascular risks. To address visual impairment, neuroprotective agents were administered in conjunction with glucocorticoids.

**Outcomes:**

Subsequent to the interventions, the patient’s chest pain and dyspnea were promptly alleviated, while the diminished visual acuity showed gradual improvement. The patient was discharged on the 12th day post-admission and exhibited continued recovery of visual function at the 1-month follow-up.

**Lessons:**

This case underscores the critical importance of recognizing the potential cardiovascular complications of methanol poisoning. Severe metabolic acidosis induced by methanol intoxication may serve as an important contributing factor for coronary artery spasm. Timely recognition of clinical symptoms and comprehensive multidisciplinary management strategies are vital to improving patient outcomes.

## 1. Introduction

Methanol poisoning constitutes a life-threatening condition, frequently manifesting as severe metabolic acidosis, neurological deficits, and visual impairments.^[1,2]^ Acute myocardial infarction (AMI) resulting from methanol poisoning is a rare but potentially fatal complication, with limited cases reported in the medical literature. We present a rare occupational case of inhalational methanol poisoning triggering acute coronary artery spasm (CAS)-mediated AMI, highlighting this critical cardiological complication. Methanol poisoning can lead to severe metabolic acidosis, which may trigger CAS through the activation of a calcium-activated chloride channel (CaCC) in coronary smooth muscle cells. This channel activation is likely to promote coronary hypercontractility, thereby contributing to the occurrence of vasospasm.^[3]^

## 2. Case presentation

A 46-year-old man presented to our emergency department with chest pain and dyspnea persisting for 4 hours. Meanwhile, the patient experienced dizziness and blurred vision. On admission, physical examination revealed a body temperature of 36.9°C, a respiratory rate of 22 breaths per minute, a heart rate of 78 beats per minute, and a blood pressure of 134/82 mm Hg. The patient reported a history of tobacco use and alcohol consumption, but denied any prior diagnosis of hypertension, coronary artery disease, diabetes mellitus, or other chronic medical conditions. No contributory family history of cardiovascular disorders was documented.

**Figure 1.**
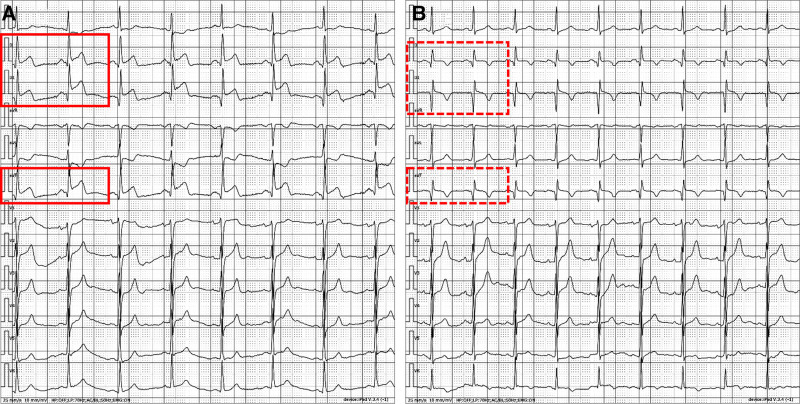
Electrocardiographic (ECG) changes. (A) Admission ECG showing ST-segment elevation in leads II, III, and aVF (red solid box). (B) Postcoronary angiography ECG revealing regression of ST-segment elevation, with formation of pathological Q waves and T-wave inversion (red dashed box).

The electrocardiogram (ECG) showed ST-segment elevation in leads II, III, and aVF (Fig. 1A). The concentration of cardiac biomarkers were elevated as follows: high-sensitivity cardiac troponin T was 252 pg/mL (normal range 0–14 pg/mL), creatine kinase was 908 U/L (normal range 50–310 U/L), creatine kinase-MB was 119 U/L (normal range 0–20 U/L), and myoglobin was 767 ng/mL (normal range 0–70 ng/mL) (Table 1). N-terminal pro-brain natriuretic peptide was normal. Additionally, arterial blood gas analysis on admission revealed severe metabolic acidosis (pH 7.12, PaCO_2_ 11 mm Hg, HCO_3_^‐^ 3.6 mmol/L, base excess ‐25.7 mmol/L) (Table 2). These findings of persistent chest pain, ST-segment elevation in the inferior leads (II, III, and aVF), and markedly elevated cardiac biomarkers collectively support the diagnosis of acute inferior ST-segment elevation myocardial infarction.

**Table 1:** Time-dependent changes of cardiac biomarkers.

Reference range	Testing time					
	0 h	2 h	1 d	2 d	3 d	6 d
hs-cTnT (0–14 pg/mL)	252	2437	2984	941	780	493
CK (50–310 U/L)	908	1713	1867	472	146	61
CK-MB (0–20 U/L)	119	247	189	50	28	20
Mb (0–70 ng/mL)	767	2611	96	46	49	36
LDH (120–250 U/L)	336	528	715	605	532	362

0 h represents admission baseline; 2 h indicates postcoronary angiography evaluation; 1–6 d denotes sequential daily measurements after admission.

CK = creatine kinase, CK-MB = creatine kinase-MB isoenzyme, hs-cTnT = high-sensitivity cardiac troponin T, LDH = lactate dehydrogenase, Mb = myoglobin.

**Table 2:** Changes in arterial blood gas results.

Reference range	Testing time					
	0 h	2 h	7 h	13 h	1 d	2 d
pH (7.35–7.45)	7.12	7.12	7.38	7.5	7.46	7.49
PaO_2_ (80–100 mm Hg)	113	144	124	106	94	122
PaCO_2_ (35–45 mm Hg)	11	10	16	23	31	30
HCO_3_^‐^ (21–28 mmol/L)	3.6	3.3	9.5	17.9	22	22.9
BE (‐3 to 3 mmol/L)	‐25.7	‐26	‐15.6	‐5.3	‐1.8	‐0.4

0 h represents admission baseline; 2 h indicates postcoronary angiography evaluation; 7 h denotes initiation of CRRT for 2 h; 13 h refers to continuation of CRRT for 8 h; 1 d signifies the 1st day post-admission with CRRT completed; 2 d represents the 2nd day post-admission.

BE = base excess, CRRT = continuous renal replacement therapy, HCO_3_⁻ = bicarbonate ion, PaCO_2_ = partial pressure of carbon dioxide in arterial blood, PaO_2_ = partial pressure of oxygen in arterial blood, pH = potential of hydrogen.

**Figure 2.**
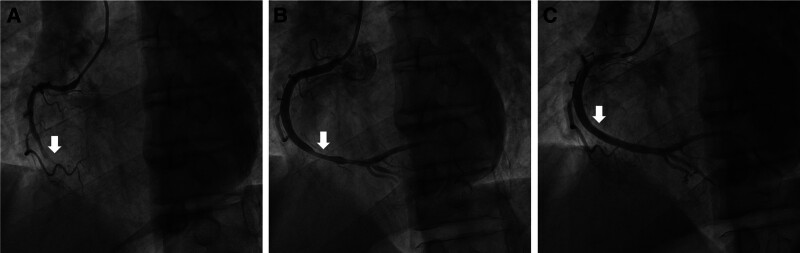
Coronary angiography. (A) Initial imaging demonstrating complete occlusion in the mid-segment of the right coronary artery (white arrow). (B) Following balloon angioplasty, occlusion was relieved, presenting as localized vascular spasm (white arrow). (C) Complete resolution of coronary artery stenosis after intracoronary administration of nitroglycerin (white arrow).

After oral administration of aspirin and clopidogrel for antiplatelet therapy, emergency coronary angiography (CAG) was performed, revealing complete occlusion of the mid-segment of the right coronary artery (Fig. 2A). Following balloon angioplasty, the occlusion was relieved, presenting as localized vascular spasm (Fig. 2B). Subsequent intracoronary nitroglycerin administration and repeated angiography demonstrated complete resolution of the right coronary artery stenosis (Fig. 2C). Notably, the CAG findings indicated that the patient’s AMI was attributed to severe CAS rather than coronary artery thrombosis. The patient was transferred to the emergency intensive care unit for further management after CAG. Although chest pain subsided post-procedure, the patient continued to experience dyspnea and reported blurred vision. Subsequent ECG revealed substantial regression of ST-segment elevation in the II, III, and aVF, accompanied by the development of pathological Q waves and T-wave inversion (Fig. 1B).

Despite aggressive rehydration and acid correction therapy, repeat arterial blood gas analysis demonstrated persistent severe metabolic acidosis (pH 7.12, PaCO_2_ 10 mm Hg, HCO_3_^‐^ 3.3 mmol/L, base excess ‐26 mmol/L) (Table 2). The anion gap was 24.6 mmol/L, which was notably elevated compared to the normal range of 8 to 16 mmol/L. Bedside ophthalmic assessment revealed reduced bilateral visual acuity (distance: counting fingers at 1 m; near: 0.25). Anterior segment examination demonstrated transparent corneas, normal anterior chambers, clear irises, and reactive pupils (3 mm diameter). Fundoscopy revealed optic discs with clearly defined margins and light red coloration (cup-to-disc ratio = 0.3; arteriole-to-venule ratio = 2:3). The foveal reflex was present, with no observable retinal hemorrhage, edema, or exudation in the posterior pole. No conjunctival injection, ocular motility restriction, or proptosis was noted. The co-occurrence of high-anion-gap metabolic acidosis and visual impairment was suggestive of methanol poisoning. A detailed occupational history revealed that the patient had been involved in pyrotechnic bright bead production during the preceding 2 days, potentially exposing him to industrial alcohol vapors containing methanol for 6 hours daily (from 09:00 to12:00 and 14:30 to 17:30). Regrettably, the methanol concentration in the work environment was not monitored. The patient presented with symptoms of dizziness, fatigue, and blurred vision 2 hours following the last exposure, and subsequently developed chest pain and dyspnea 13 hours after the final exposure. Toxicological analysis confirmed the diagnosis, revealing a blood methanol concentration of 823 μg/mL.

**Figure 3.**
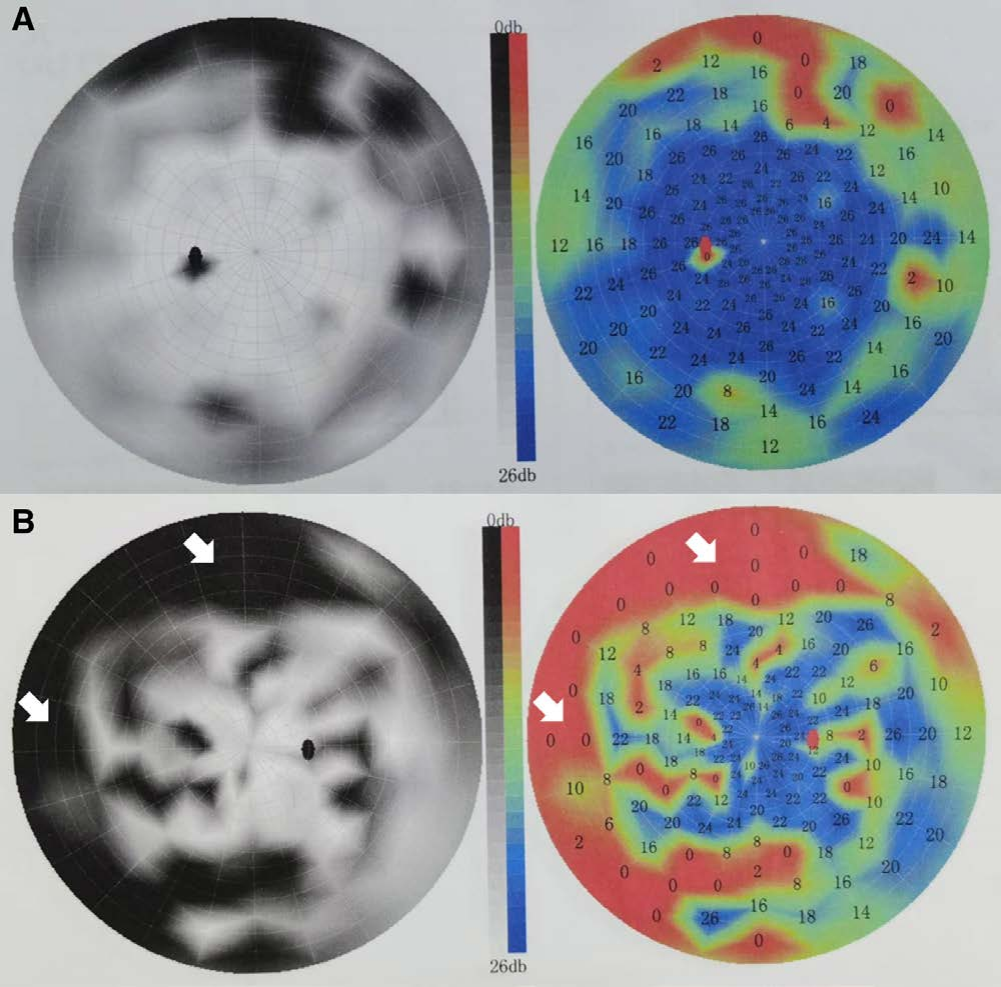
Ophthalmic visual field examination. (A) Left eye visual field. (B) Right eye visual field. White arrow indicates visual field defect in the right eye.

Given the patient’s condition and diagnosis, a single session of continuous renal replacement therapy (CRRT) was immediately initiated. Following CRRT, the patient’s metabolic acidosis rapidly improved (Table 1), along with significant alleviation of dyspnea. Fomepizole was not used owing to its unavailability in the region. Additionally, ethanol was not administered because of its potential cardiovascular risks, particularly in the context of the patient’s AMI. To mitigate potential optic nerve damage and improve visual prognosis, a combination of neuroprotective agents and glucocorticoid therapy was administered. A follow-up blood toxicology screening performed 2 days later confirmed the complete elimination of methanol. Five days post-admission, the patient underwent a comprehensive ophthalmological evaluation, which revealed visual acuity of 0.6 in the left eye and 0.4 in the right eye, with notable constriction of the right eye’s visual field (Fig. 3). Both fundus examination and optical coherence tomography yielded unremarkable results. These findings were indicative of methanol-induced toxic optic neuropathy. Consequently, the patient was referred to the ophthalmology department for specialized vision restoration treatment and discharged on the 12th day after admission. Following ophthalmological examination at 1-month follow-up, the patient’s visual acuity had improved to 0.8 in the left eye and 0.6 in the right eye. Optical coherence tomography revealed maintained retinal nerve fiber layer thickness and a normal optic cup-to-disc ratio.

## 3. Discussion

This case report describes a rare and significant instance of CAS and AMI associated with methanol poisoning, emphasizing the critical interplay between toxicological exposure and cardiovascular events. The clinical challenges in this case, including acute chest pain, dyspnea, severe metabolic acidosis, and visual impairment, highlight the importance of prompt recognition and timely management of methanol poisoning, especially when accompanied by cardiovascular complications.

The clinical manifestations of methanol poisoning are diverse.^[4,5]^ The early signs and symptoms are typically nonspecific and may include gastrointestinal symptoms such as nausea, vomiting, and abdominal pain, as well as neurological symptoms such as dizziness, headache, somnolence, and fatigue. As acidosis progresses, visual symptoms including blurred vision and visual impairment may emerge. Furthermore, there is a possibility of an exacerbation of central nervous system symptoms, which may result in memory loss, agitation, confusion, and even coma. Methanol is a highly toxic substance that causes severe damage to the central nervous system including optic nerve. Its metabolite, formic acid, has been observed to bind to mitochondrial cytochrome c oxidase, thereby inhibiting mitochondrial oxidative phosphorylation, and consequently causing tissue oxygen utilization disorders and neuronal damage.^[6]^

The prevalence of long-term visual impairment in patients with methanol poisoning may be underestimated. A longitudinal study involving 50 patients with methanol poisoning found that 40% exhibited persistent visual sequelae during follow-up, including an 8% rate of blindness.^[7]^ Among these patients, 38% displayed abnormalities in the retinal nerve fiber layer, while 40% demonstrated abnormal visual evoked potentials, indicating widespread functional alterations in visual pathways following methanol exposure.^[7]^ The patient in our case exhibited favorable short-term recovery, with visual acuity improving to 0.8 in the left eye and 0.6 in the right eye at the 1-month follow-up, along with preserved retinal nerve fiber layer thickness and normal cup-to-disc ratios. However, the integrity of the retinal nerve fiber layer at this early stage does not preclude the possibility of delayed degeneration, as subclinical axonal loss may manifest months after exposure. Therefore, a definitive long-term visual prognosis in this case necessitates extended ophthalmic surveillance.

Despite the increased prevalence of studies on methanol poisoning, the majority have concentrated on visual impairment and central nervous system damage. There is a paucity of research on cardiovascular complications of methanol poisoning, such as CAS and AMI. CAS is a cause of myocardial infarction with nonobstructive coronary arteries.^[8]^ ECG manifestations of CAS include ST-segment elevation or depression, negative U waves, and T-wave abnormalities.^[9]^ In the present case, the initial presentation of ST-segment elevation in leads II, III, and aVF, along with elevated cardiac biomarkers, was consistent with an inferior wall AMI. However, angiographic findings ultimately revealed that the AMI was caused by CAS rather than thrombus obstruction, highlighting the importance of considering CAS in similar clinical presentations. Approximately 10% of patients experiencing a myocardial infarction do not exhibit obstructive coronary artery disease.^[10]^ These individuals are at a reduced risk of recurrent cardiovascular events compared to those with myocardial infarction accompanied by obstructive coronary artery disease.^[10]^ In the management of AMI, particularly during coronary intervention procedures, dual antiplatelet therapy is commonly employed to mitigate the risk of cardiovascular events and enhance long-term outcomes.^[11]^ However, a study by Lindahl et al.^[12]^ found that dual antiplatelet therapy had a neutral impact on major adverse cardiac events in patients with myocardial infarction without obstructive coronary artery disease, with a hazard ratio of 0.90 and 95% confidence intervals ranging from 0.74 to 1.08 during a 1-year follow-up period. Currently, there is a paucity of clinical trials investigating the use of antiplatelet therapy in patients experiencing AMI secondary to methanol poisoning. Existing studies have indicated that methanol poisoning can precipitate cerebral hemorrhage, particularly affecting the bilateral putamina.^[13]^ Marumo et al^[14]^ demonstrated that toxic concentrations of formic acid (≥0.01%), a key metabolite in methanol intoxication, inhibit platelet aggregation, likely via extracellular acidosis and reduced Ca^2+^ entry into platelets. This formic acid-induced platelet dysfunction can consequently lead to a bleeding tendency in methanol poisoning, potentially contributing to the pathogenesis of intracranial hemorrhage. The use of antiplatelet agents in patients with methanol poisoning could potentially increase the risk of intracranial hemorrhage. Consequently, clinicians must carefully balance the potential benefits of mitigating thrombotic events against the heightened risk of hemorrhage when considering pre-catheterization antiplatelet therapy in patients with methanol poisoning complicated by AMI. Decisions should be individualized, taking into account factors such as the severity of acidosis, neurological status, platelet count, and other relevant clinical parameters.

In our case, metabolic acidosis resulting from methanol poisoning may serve as a significant predisposing factor for acute CAS. Similarly, Vivas et al^[15]^ reported a case of chronic metabolic acidosis associated with recurrent episodes of vasospastic angina. These episodes were characterized by chest pain, diaphoresis, and dyspnea. During each episode, ECGs showed ST-segment elevation in the inferior wall leads, while subsequent CAG did not reveal significant coronary artery stenosis. Notably, exacerbation of metabolic acidosis was observed during all 3 episodes. This finding suggests a potential association between severe metabolic acidosis and the triggering of CAS. The pathophysiological mechanism underlying acidosis-induced coronary artery spasm may be associated with the enhanced activity of the TMEM16A/anoctamin 1 (ANO1), a CaCC, in coronary arterial smooth muscle cells. Guo et al^[3]^ demonstrated that, in comparison to other arterial beds, rat coronary arterial smooth muscle cells exhibit elevated expression levels of TMEM16A/ANO1, as well as increased CaCC currents and membrane depolarization in response to extracellular acidosis. Acidosis leads to a reduction in intracellular chloride, facilitating chloride efflux via CaCC and initiating a positive feedback loop that involves enhanced intracellular Ca^2+^ release and extracellular Ca^2+^ influx through L-type voltage-gated Ca^2+^ channels, ultimately resulting in the hypercontractility of coronary arteries. Importantly, the application of chloride channel blockers, sarcoplasmic reticulum Ca^2+^ release inhibitors, L-type voltage-gated Ca^2+^ channel blockers, and ANO1 antibodies all mitigate acidosis-induced rat coronary artery constriction and the associated electrophysiological alterations. This research suggests that the increased activity of CaCC in coronary artery smooth muscle cells, coupled with enhanced calcium mobilization, plays a crucial role in the hypercontraction induced by acidosis.

The management of methanol poisoning requires a comprehensive, multidisciplinary approach aimed at correcting metabolic acidosis, eliminating toxic metabolites, and minimizing potential complications. Initial interventions typically involve aggressive fluid resuscitation and the administration of specific antidotes, such as fomepizole and ethanol.^[16]^ Methanol metabolism is predominantly facilitated by the hepatic enzyme alcohol dehydrogenase. Fomepizole acts as a competitive inhibitor of alcohol dehydrogenase, thereby preventing the formation of methanol metabolites.^[16]^ Alternatively, ethanol, a competitive substrate for alcohol dehydrogenase, may be administered to inhibit methanol metabolism.^[16]^ Nonetheless, ethanol administration is associated with unpredictable pharmacokinetics and may induce alterations in mental status, hypoglycemia, pancreatitis, and an elevated risk of cardiovascular diseases. For this patient, fomepizole was not utilized due to its unavailability, as it is not a marketed drug in our region and could not be acquired through emergency procurement channels during the acute treatment phase. Furthermore, ethanol was not administered because of its potential cardiovascular risks in the context of the patient’s AMI. Hemodialysis is an effective intervention for the removal of methanol and its metabolites, as well as for the rapid correction of metabolic acidosis, thereby constituting a crucial treatment modality for methanol poisoning.^[17]^ Current clinical guidelines advocate for the consideration of hemodialysis in patients presenting with a serum methanol concentration of at least 50 mg/dL (15.6 mmol/L), significant acidemia, or visual manifestations.^[16]^ In cases where patients exhibit hemodynamic instability, severe cerebral edema, or are unable to undergo hemodialysis, CRRT is recommended as an alternative therapeutic approach. In the present case, the prompt initiation of CRRT facilitated the rapid correction of metabolic acidosis and significant symptomatic improvement. Furthermore, the combination of neuroprotective agents and glucocorticoids likely played a crucial role in the patient’s recovery of visual function. Follow-up evaluations showing significant visual improvement underscore the importance of interdisciplinary approaches in managing complex cases involving multiple organ systems.

This case report possesses several limitations that warrant Acknowledgments. Firstly, as a singular case study, the findings are not generalizable to the wider population of patients with methanol poisoning. The distinct presentation of CAS and AMI may not be indicative of all cases of methanol toxicity. Secondly, the detailed pathophysiological mechanism linking severe metabolic acidosis to CAS remains speculative and necessitates further exploration through comprehensive experimental studies. Additionally, the long-term cardiovascular and visual sequelae of methanol poisoning were not thoroughly assessed beyond the 1-month follow-up period. Future multicenter studies with larger patient cohorts are essential to validate and expand upon the observations documented in this case report.

## 4. Conclusion

Methanol poisoning is marked by metabolic acidosis, optic neuropathy, and central nervous system damage. This case highlights that severe methanol poisoning can lead to CAS and AMI, primarily due to significant metabolic acidosis. Clinicians should promptly assess for methanol exposure in patients with AMI, unexplained high-anion-gap metabolic acidosis, and visual impairment, especially in industrial settings. Effective management involves acidosis correction, fomepizole administration (if available), hemodialysis or CRRT, and neuroprotective strategies, such as glucocorticoids and neurotrophic agents, to address potential optic neuropathy.

## Author contributions

**Data curation:** Dun Ao.

**Formal analysis:** Shu Zhou.

**Funding acquisition:** Jinjun Li.

**Investigation:** Dun Ao.

**Writing – original draft:** Jinjun Li, Shu Zhou.

**Writing – review & editing:** Jinjun Li.
